# Case report: Discovery of tuberculosis caused by *Mycobacterium bovis* in free-ranging vervet monkeys in the Greater Kruger Conservation Area

**DOI:** 10.3389/fvets.2024.1460115

**Published:** 2024-12-10

**Authors:** Lin-Mari de Klerk-Lorist, Michele A. Miller, Emily P. Mitchell, Rudolf Lorist, D. Schalk van Dyk, Nomkosi Mathebula, Louise Goosen, Rebecca Dwyer-Leonard, Giovanni Ghielmetti, Elizabeth M. Streicher, Tanya J. Kerr

**Affiliations:** ^1^State Veterinary Office & Laboratory, DALRRD, Kruger National Park, Skukuza, South Africa; ^2^SAMRC Centre for Tuberculosis Research, Division of Molecular Biology and Human Genetics, Faculty of Medicine and Health Sciences, Stellenbosch University, Cape Town, South Africa; ^3^Department of Paraclinical Sciences and Centre for Veterinary Wildlife Research, Faculty of Veterinary Science, University of Pretoria, Pretoria, South Africa; ^4^Bushbuckridge South State Veterinary Office, Thulamahashe, South Africa; ^5^Section of Veterinary Bacteriology, Institute for Food Safety and Hygiene, Vetsuisse Faculty, University of Zurich, Zurich, Switzerland

**Keywords:** animal tuberculosis, *Chlorocebus pygerythrus*, *Mycobacterium bovis*, one health, vervet monkeys, wildlife

## Abstract

Animal tuberculosis (TB) has been reported in several wildlife species in the Greater Kruger Conservation Area (GKCA), South Africa. This report describes the discovery of clinical tuberculosis, caused by *Mycobacterium bovis* (*M. bovis*), in free-ranging vervet monkeys (*Chlorocebus pygerythrus*). The “One Health” concept is especially relevant to TB since this is a multi-host disease with zoonotic potential and is endemic in GKCA. Vervet monkeys have become habituated to humans in tourist areas and may be a source of infection through close contact. Indirect transmission of *M. bovis* through environmental sources has also been suspected to present a risk of spread between host species. Clinically diseased monkeys present in two tourist areas in the GKCA, that died (*n* = 1) or were euthanized (*n* = 5), were submitted for diagnostic necropsies. The presence of pathological lesions, Ziehl-Neelsen-stained impression smears, Xpert® MTB/RIF Ultra (GXU) assay, mycobacterial culture and speciation by genomic regions of difference PCR, were used to confirm the diagnosis of *M. bovis* infection in these monkeys. The finding of multiple cases necessitates further investigation of TB in monkey troops living within the GKCA tourist areas to determine the source of infection and assess the risk of transmission to other animals and humans.

## 1 Introduction

Vervet monkeys (*Chlorocebus pygerythrus*) occupy diverse habitats across southern and eastern Africa, predominantly open woodland and savannahs, but also commonly found in several peri-urban areas (1, 2). As habitats are modified by development, this can result in competition between wildlife and humans for resources and space. Due to their flexibility in foraging strategies and absence of major threats, vervet monkeys often come into conflict with humans, because of shared human-dominated landscapes, which also holds true for game reserves and parks (3, 4). Vervet monkeys are therefore exposed to anthropogenic risks, including infectious diseases (5). However, they may also present zoonotic threats to humans, especially in areas where endemic diseases are present (6, 7).

There are numerous reports of *Mycobacterium tuberculosis* outbreaks in captive and free-ranging Old World primates, usually that have either direct or indirect contact with humans (8–10), including free-ranging Chacma baboons (*Papio ursinus*) in the Cape Peninsula, South Africa (11). Natural infections with two different *M. tuberculosis* strains were found in two vervet monkeys at a rehabilitation centre in South Africa following deaths of several individuals (12, 13). Generally, Old World monkeys kept under intensive captive conditions appear to be susceptible to infection by any route and can develop fulminating, fatal disease (14, 15).

In comparison, there is less knowledge about *Mycobacterium bovis* infection in Old World monkeys. *Mycobacterium bovis* infection and disease, as well as infection with other MTBC, such as *Mycobacterium orygis* and *Mycobacterium caprae*, has been sporadically reported in captive non-human primates (16–21). However, studies of *M. bovis* infection in free-ranging African primates are more limited, although they have been focused on *M. bovis* endemic areas. For example, wild chacma baboons were screened at a human-wildlife interface in the Kafue Flats, Zambia, where *M. bovis*-infected cattle (*Bos taurus*) and lechwe (*Kobus leche*) populations were present. Gross lesions consistent with tuberculosis (TB) in the lungs and associated lymph nodes were found in four adult male baboons (22). The *M. bovis* spoligotypes found in these baboons, SB0120, was the same strain reported in humans, livestock, and wildlife in the Kafue ecosystem. Opportunistic sampling of wildlife carcasses in Ruaha ecosystem, Tanzania, identified *M. bovis* from tissues of two vervet monkeys and one yellow baboon (*Papio cynocephalus*), although no gross lesions were observed (23). Spoligotyping of the isolates revealed the same *M. bovis* strain, SB0133, found in local livestock. These cases highlight the risk of infection to non-human primates in ecosystems with other *M. bovis* infected hosts.

Animal TB, caused by *M. bovis* infection, has been diagnosed in various wildlife species in the Greater Kruger Conservation Area (GKCA) in South Africa, since the first detection in 1990, and is now considered endemic (24, 25). Cases in non-human primates have included a TB outbreak in 1996 in a chacma baboon troop that frequented the Skukuza tourist area in GKCA (26). A second outbreak was recorded in 2010 in the same area, within a baboon troop also displaying unusual roosting behavior, by accessing a closed workshop roof at night. A program of testing and euthanasia was applied, as previously described by Keet et al. (26), and the disease was eradicated in the Skukuza baboon troop (27). However, despite continued surveillance using necropsy findings, no cases of *M. bovis* or *M. tuberculosis* infection have previously been found in vervet monkeys, although troops occupy the same areas as the baboons. Therefore, this case series describes the first report of clinical TB in free-ranging vervet monkeys in the GKCA, South Africa.

## 2 Case descriptions and clinical findings

Between June 2023 and May 2024, free-ranging vervet monkeys, found in tourist areas in Skukuza (*n* = 5) and a private lodge in the Sabie Game Reserve (*n* = 1), were reported with signs of depression, weakness, and emaciation. Both these areas are part of the GKCA but approximately 12 km apart, and are considered endemic for *M. bovis* (24, 25, 28). The case in Sabie Game Reserve died after observing clinical signs. The animals in Skukuza were captured using a baited trap and then immobilized by pole syringe or plastic projectile dart, using a combination of tiletamine-zolazepam [Zoletil®; Virbac RSA, (Pty) Ltd., Centurion, South Africa] and ketamine [Kyron Laboratories (Pty) Limited, Benrose, South Africa], both at 10 mg/kg IM. Animals were humanely euthanized while anesthetized, with sodium pentobarbitone (Euthapent; Kyron Laboratories) at 200 mg/kg IV, followed by a thorough postmortem examination and tissue sample collection. This included spleen, lung, and lymph nodes (pooled head, thoracic, abdominal, and peripheral lymph nodes), as well as other organs with lesions consistent with TB. Fresh samples were frozen, and a second set of tissues was stored in 10% buffered formalin for histopathological examination.

## 3 Diagnostic assessment

Frozen tissues were processed in a BSL3 laboratory for initial screening with a rapid qPCR assay (GXU; Xpert MTB/RIF Ultra; Cepheid Sunnyvale, CA, USA) for detection of *Mycobacterium tuberculosis* complex DNA, and mycobacterial culture using the BD BACTEC™ MGIT™ 960 system (Becton Dickinson, Franklin Lakes, NJ, USA), as previously described (29). Cultures positive for growth were genetically speciated by genomic regions of difference PCR (30) to confirm infection with *M. bovis*.

**Figure 1:**
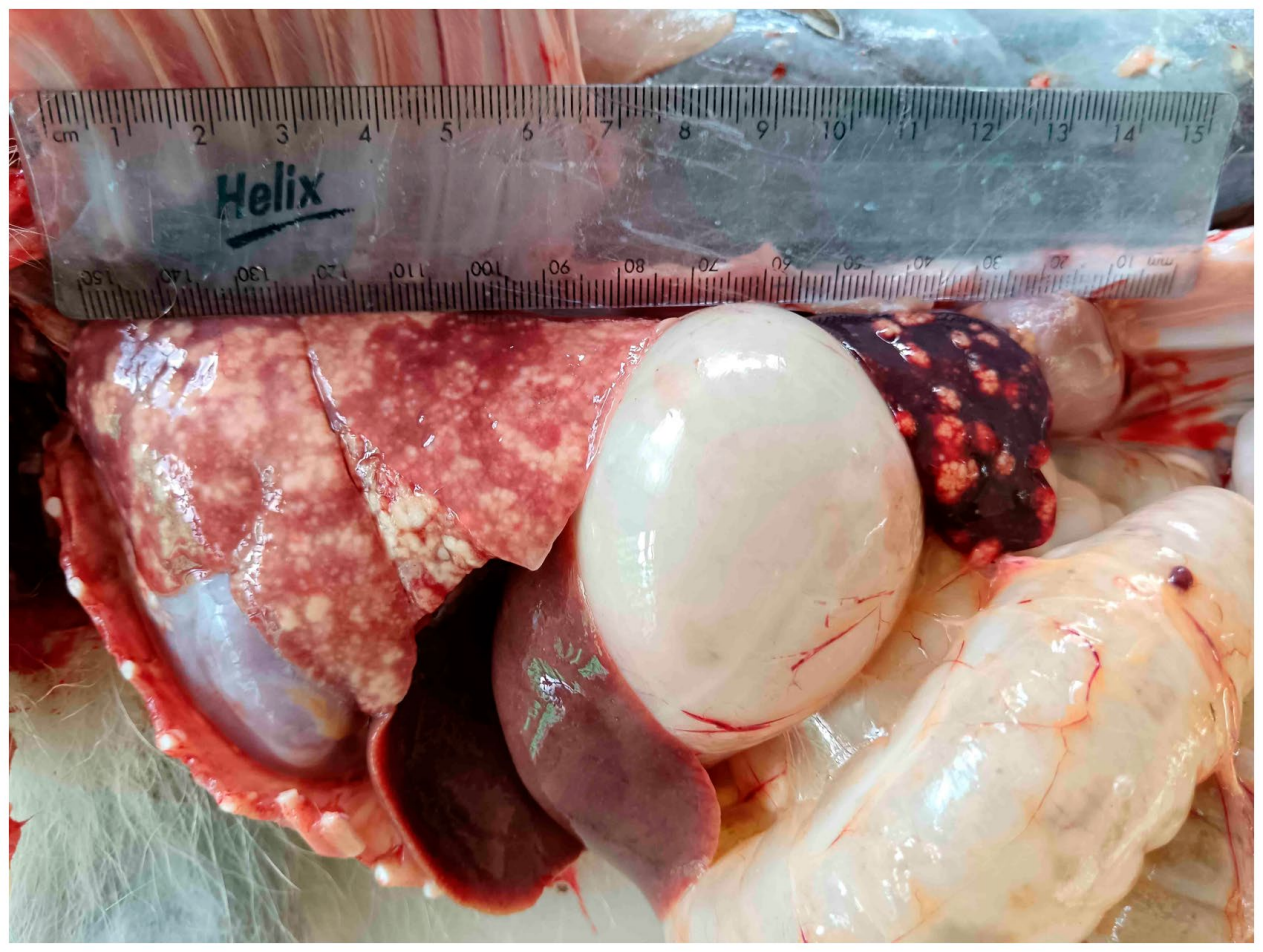
Macroscopic lesions in lung and spleen of a juvenile male vervet monkey (*Chlorocebus pygerythrus*), that was euthanized in 2024 from the Skukuza tourist camp in Greater Kruger Conservation Area, South Africa. Lesions were similar to other tuberculosis cases in vervet monkeys in this area, associated with *Mycobacterium bovis* infection. *Mycobacterium tuberculosis* complex DNA was detected in this case using qPCR (Cepheid Xpert MTB/RIF Ultra qPCR assay).

**Figure 2:**
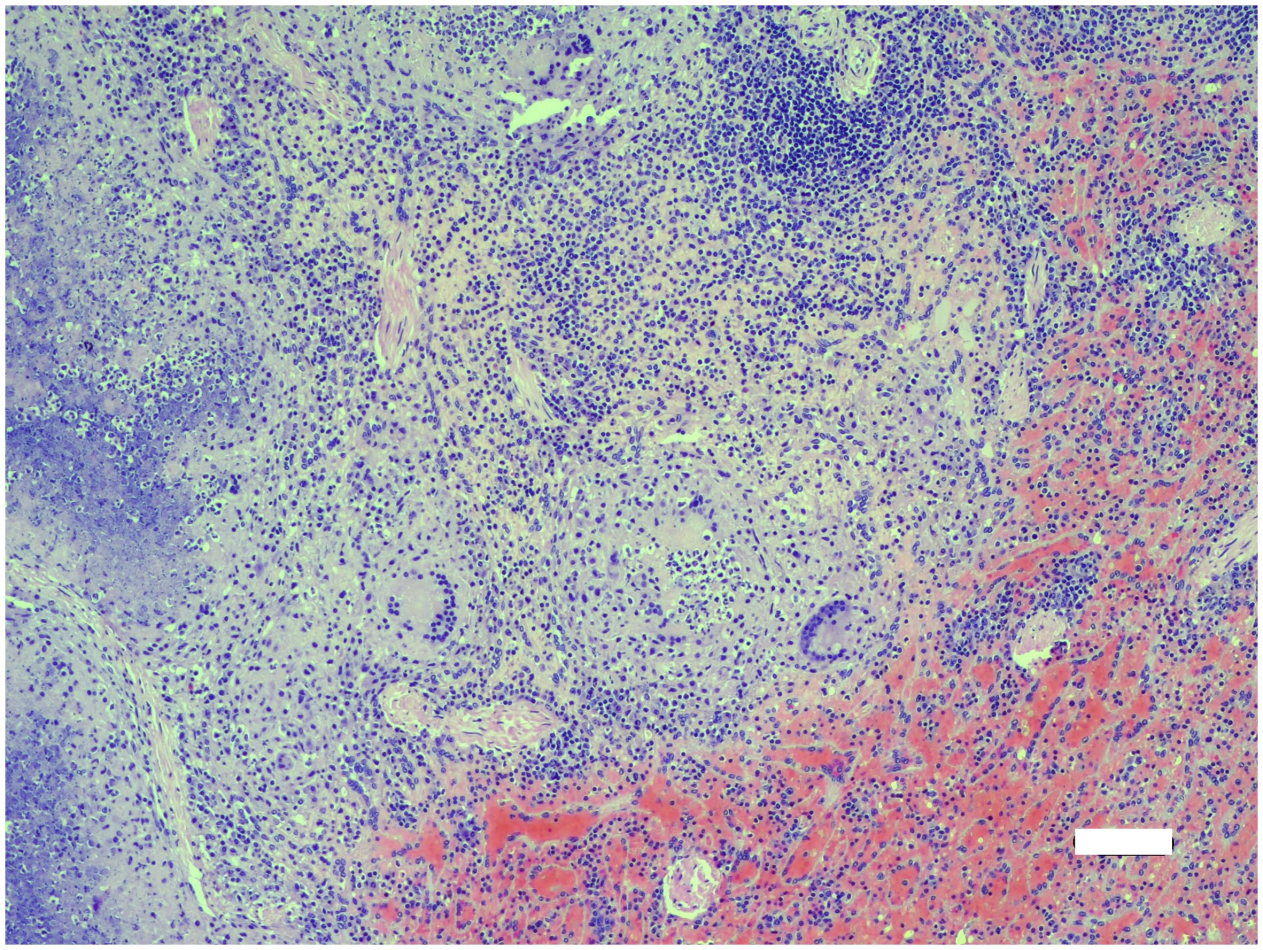
Microscopic lesions in spleen of adult female vervet monkey (*Chlorocebus pygerythrus*); necrogranulomatous splentitis with large multinucleate giant cells and marked splenic congestion (lower right). H&E x100. Bar = 100 μm.

**Figure 3:**
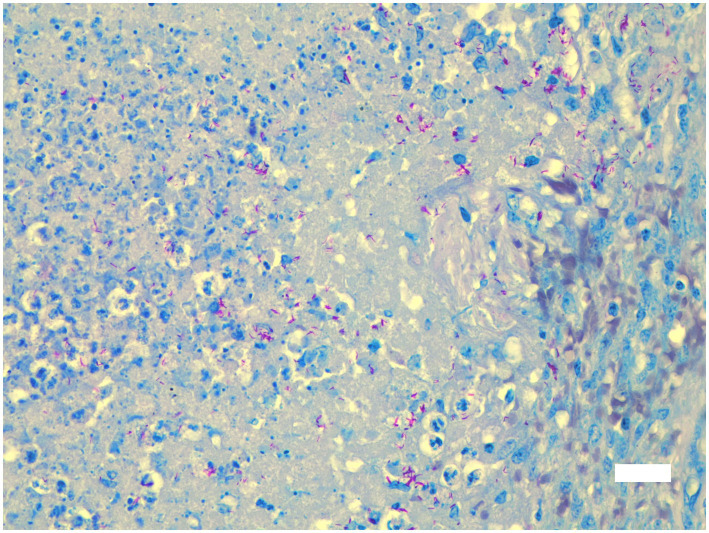
Microscopic lesions in spleen of adult female vervet monkey (*Chlorocebus pygerythrus*); necrogranulomatous splenitis with large numbers of fine acid-fast (red) bacilli in necrotic debris. Ziehl Neelsen x 400. Bar = 30 μm.

All six vervet monkeys (2 males, 4 females, including 3 adults >2 years of age; 2 sub-adults 1–2 years; 1 juvenile <1 year) had gross lesions consistent with TB in multiple organs, with spleen and lung lesions present in all six clinical cases (Table 1). Lungs were generally bilaterally affected with multiple necrogranulomatous lesions, varying from 1 mm to almost confluent throughout the parenchyma (Figure 1). The dorso-caudal lung lobes were more severely affected compared to the middle and cranial lobes, while only one adult female monkey had a unilateral left caudal tuberculous pneumonia. Necrogranulomatous lesions were also a common feature in the spleens of diseased monkeys, with multiple granulomas varying in size from 1 to 8 mm in diameter (Figures 2, 3). Impression smears from lung and spleen of each case were used for Ziehl-Neelsen staining. All six cases were confirmed with *M. bovis* infection, based on mycobacterial culture and speciation, GXU, macro- and micro- histopathological techniques. Results are summarized in Table 1.

**Table 1:** Summary of demographic information and *Mycobacterium bovis* test results of six free-ranging vervet monkeys (*Chlorocebus pygerythrus*) sampled in the Greater Kruger Conservation Area, South Africa.

Case	Age	Sex	Sample date	Clinical signs	Macroscopic lesions present	Histological lesions present	Ziehl-Neelsen stain (cytology)	GXU®^#^	Speciation
1	Sub-adult	Female	28 Jun 2023	Depressed, cough, emaciated, weakness, incoordination	S, P, M, L	S, P, K	++	MTBC detected – medium; no RIF resistance	*M. bovis*
2	Adult	Female	1 Aug 2023	Depressed, emaciated, weakness	S, P, H, M	NA	+++	MTBC detected – high; no RIF resistance	*M. bovis*
3	Adult	Female	1 Feb 2024	Depressed, emaciated, weakness, visibly swollen lymph nodes	S, P, M, A	S (only tissue examined)	++	MTBC detected – high; no RIF resistance	*M. bovis*
4	Juvenile	Male	30 Mar 2024	Depressed, emaciated, weakness, visibly swollen lymph nodes, incoordination, dyspnoea	S, P, H, M, A, L	S, P, H, E	*+++*	MTBC detected – high; no RIF resistance	*M. bovis*
5	Adult	Female	30 Apr 2024	Depressed, emaciated, weakness, dyspnoea incoordination	S, P, M	S, P, K	+	MTBC detected – high; no RIF resistance	*M. bovis*
6	Sub-adult	Male	4 May 2024	Depressed, emaciated, weakness, dyspnoea	S, P, H, M, K	S, P, L, M, K	+	MTBC detected – medium; no RIF resistance	*M. bovis*

S, Spleen; P, Lungs & pulmonary lymph nodes (lnn), H, Head lnn; M, Mesenteric lnn; A, Mammary/axillary lnn; L, Liver; K, Kidney; E, enteritis; NA, not available; GXU, Cepheid Xpert MTB/RIF Ultra qPCR assay; RIF, rifampicin; *histopathologic analyses performed; #the levels of MTBC detected (very low/low/medium/high) by the Cepheid Xpert MTB/RIF Ultra qPCR assay were based on preprogrammed rpoB cycle threshold (Ct) values; very low (Ct > 28), low (Ct 22–28), medium (Ct 16–22) or high (Ct < 16).

+ 0–50 AFB per field; ++ 50–100 AFB per field; +++ > 100 AFB per field.

Histopathological examination was performed on selected tissues from five monkeys (cases 1, 3–6; no formalin fixed tissues were available for case 2). Spleen, lung and/or lymph nodes were virtually effaced by unencapsulated coalescing necrogranulomas in all five monkeys. Additional lesions included mild to moderate necrogranulomatous nephritis (cases 1, 4–6), hepatitis (cases 5, 6) and enteritis (case 4), often with numerous multinucleate giant cells. Only spleen was submitted from case 3. Mild to moderate lymphohistiocytic perivascular interstitial pneumonia was also present in the four monkeys for which lung was submitted. Rare acid-fast organisms (Ziehl-Neelsen) were seen in multinucleate giant cells, but organisms were more common in necrotic debris and macrophages in splenic granulomas, macrophages in neutrophil-rich bronchiolar exudate and in tissue impression smears (Table 1).

*Mycobacterium bovis* infection was confirmed by culture and speciation from multiple tissues in all six vervet monkeys. The tissues with positive culture isolation included lung, liver, spleen, kidney, and thoracic, mesenteric, and head lymph nodes. In addition, urine was collected from the bladder post-mortem from case 6 and confirmed to contain viable bacilli. Similar to culture, samples from multiple tissues were positive using the GXU, but not for the urine sample. However, none of the samples showed rifampicin resistance, based on the GXU readout.

## 4 Discussion

This report describes the first known outbreak of clinical TB, caused by *M. bovis* infection, in free-ranging vervet monkeys in the GKCA, South Africa. However, susceptibility of non-human primates to TB, caused by *M. tuberculosis* infection, has been recognized for nearly five decades (31–33). The similarities of TB in non-human primates and humans, especially macaques, has led to their use as an animal model for investigating TB pathogenesis, drugs, and vaccines (34, 35). However, in an experimental infection study, vervet monkeys appeared to be more susceptible to *M. tuberculosis*, than rhesus (*Macaca mulatta*) or cynomolgus (*Macaca fascicularis*) macaques (35).

The wildlife populations in the GKCA and Hluhluwe-iMfolozi Park (HiP), in KwaZulu-Natal province (South Africa), are considered endemic for *M. bovis* (24). Although the primary *M. bovis* maintenance host in both areas is African buffalo (*Syncerus caffer*), spillover to numerous other species, including lions (*Panthera leo*), greater kudu (*Tragelaphus strepsiceros*), white rhinoceros (*Ceratotherium simum*), and banded mongooses (*Mungos mungo*), has been reported (9, 36). Cases of TB in chacma baboons have occurred in GKCA (26, 37) and HiP (24). Prevalence during the 1996 outbreak reached approximately 50%, with all 14 *M. bovis* isolates sharing the same genotype (26). Infected baboons exhibited granulomatous pneumonia with lesions also found in pulmonary and mesenteric lymph nodes, and spleen. Similarly, all six vervet monkeys in the current case series had extensive macroscopic lesions in the lungs, pulmonary lymph nodes, spleen, and mesenteric lymph nodes. Lesions in the spleen and mesenteric lymph nodes suggest that monkeys were infected through the oral route. Pulmonary lesions may represent potential aerosol transmission but have also been found in animals infected with *M. bovis* orally (38, 39). Although a source was not identified in either the baboon or the current vervet monkey outbreaks, transmission of *M. bovis* from other members of the troop or wildlife species in the GKCA may have occurred either through direct contact or potentially indirectly from exposure to contaminated environments or scavenging (26, 27). Further investigations of potential sources are required since observations of the prior behavior of affected monkeys was limited.

The relatively high levels of mycobacteria in the lungs of the vervet monkeys, detected in Ziehl-Neelsen stained impression smears and by GXU, suggest that these animals were potentially excreting *M. bovis*. The finding of viable *M. bovis* in urine from case 6 demonstrates that these monkeys could be shedding and pose a risk of further transmission. Studies in other species have linked development of generalized infection and presence of large lesions with potential excretion of *M. bovis* (40, 41). The social nature of vervet monkeys supports pathogen transmission within troops (42). In addition, since these animals shared environments with tourists and staff, including raiding outdoor guest tables and staff dwellings, there is a potential risk of zoonotic transmission through direct contact with infected monkeys or indirectly through excretions (26, 43). Vervet monkey bites in tourist areas in GKCA average about 37 reported occurrences per year, with two bites occurring in June 2024, during the TB outbreak [S. Midzi, J. Dabrowski, L. Mdletshe, pers. comm]. This is a significant concern, especially in South Africa where there is a high burden of HIV-AIDS in the human population (44). However, there is limited zoonotic TB surveillance in South Africa (45). This highlights the need to study prevalence in vervet monkey populations to assess the risk of transmission to other animals and humans. Active surveillance could include tuberculin skin testing, interferon gamma release assay, and direct detection of MTBC in secretions, such as respiratory fluids and feces. Therefore, with the discovery of TB in vervet monkeys in GKCA, further surveillance and efforts to control spread should be prioritized at interfaces between wildlife and humans in *M. bovis* endemic areas.

## 5 Conclusion

The TB outbreak in the vervet monkey population inside the Skukuza Rest Camp and a private lodge was surprising and alarming. Given the potential for human interaction in areas such as restaurants, shops, camping sites, dust bins, and kitchen facilities, an *M. tuberculosis* outbreak would have been more likely, especially considering the available published data on the susceptibility of non-human primates to the human pathogen. However, vervet monkey behavior has not been associated with scavenging (46), as was reported for baboons during the *M. bovis* outbreaks in the Skukuza area (26, 37). In the absence of clinical TB in the local baboon population, there must be an alternative epidemiological link to the source of infection in the vervet monkeys, such as exposure to *M. bovis* infected banded mongoose or warthog, also found in the same areas (25, 36). The severity of lesions and clinical signs in affected monkeys suggest that *M. bovis* may have spread within the troop. Future studies should include whole genome sequencing of *M. bovis* isolates to investigate epidemiologic links between these cases and to other hosts in the GKCA.

## Data Availability

The original contributions presented in the study are included in the article/supplementary material, further inquiries can be directed to the corresponding author.
